# Real-World Evidence of Growth Improvement in Children 1 to 5 Years of Age Receiving Enteral Formula Administered Through an Immobilized Lipase Cartridge

**DOI:** 10.3390/nu18020287

**Published:** 2026-01-16

**Authors:** Alvin Jay Freeman, Elizabeth Reid, Terri Schindler, Thomas J. Sferra, Barbara Bice, Ashley Deschamp, Heather Thomas, David P. Recker, Ann E. Remmers

**Affiliations:** 1 Department of Pediatrics, The Ohio State University College of Medicine, Columbus, OH 43210, USA; 2 Division of Gastroenterology, Hepatology & Nutrition, Department of Pediatrics, Nationwide Children’s Hospital, Columbus, OH 43205, USA; 3 Department of Clinical Nutrition, Cystic Fibrosis Center, Division of Pulmonary and Sleep Medicine, Children’s Hospital of Philadelphia, Philadelphia, PA 19104, USA; ekr2020@gmail.com; 4 Division of Pediatric Pulmonology, University Hospitals Rainbow Babies and Children’s Hospital, Cleveland, OH 44106, USA; terri.schindler@uhhospitals.org; 5 Division of Pediatric Gastroenterology, Hepatology and Nutrition, Department of Pediatrics, UH Rainbow Babies & Children’s Hospital, Case Western Reserve University School of Medicine, Cleveland, OH 44106, USA; thomas.sferra@uhhospitals.org; 6 Nebraska Regional Pediatric Cystic Fibrosis Center at Children’s Nebraska, Omaha, NE 68114, USA; bbice@nebraskamed.com (B.B.); adeschamp@childrensnebraska.org (A.D.); hthomas@childrensnebraska.org (H.T.); 7 Department of Pediatrics, University of Nebraska Medical Center College of Medicine, Omaha, NE 68198, USA; 8 Alcresta Therapeutics, Inc., Waltham, MA 02453, USA; drecker@alcresta.com (D.P.R.); aremmers@alcresta.com (A.E.R.)

**Keywords:** fat malabsorption, enteral nutrition, lipase, pediatric, cystic fibrosis, short bowel syndrome

## Abstract

Background/Objectives: RELiZORB immobilized lipase cartridge (ILC) is a single-use digestive enzyme cartridge that connects in-line with enteral feeding circuits to hydrolyze triglycerides in enteral formulas. It is cleared by the FDA for pediatric and adult use. Limited data have been published regarding the effect of ILC use on growth in children younger than 5 years of age. Methods: We performed a retrospective evaluation of real-world data extracted from a third-party reimbursement program database. All patients in the program database who initiated ILC use with enteral formula when 1 to 4 years of age between 2019 and 2023 were included. Baseline and follow-up weight, height/length, and body mass index (BMI) data were collected for up to 12 months. Results: A total of 186 patients from 90 clinics in the United States were included. A subset (143 patients) with baseline and follow-up growth measurements was included in the efficacy analysis population; 76% were diagnosed with cystic fibrosis. Mean weight and BMI z-scores improved significantly (0.63 [*p* < 0.001] and 0.53 [*p* = 0.006], respectively) from baseline to 12 months after initiation of ILC use. Significant improvement in the mean weight z-score was observed after 3 months. Among people with cystic fibrosis (pwCF) who initiated ILC use when 2 to 4 years of age, those with a BMI ≥ 50th percentile increased from 22% at baseline to 43% after 12 months (*p* = 0.021). Improvement in weight-for-length was also observed in 1-year-old pwCF. Conclusions: Real-world evidence showed that initiation of ILC use was associated with significant improvements in mean weight and BMI z-scores among young children.

## 1. Introduction

Although exocrine pancreatic insufficiency (EPI), as seen in patients with cystic fibrosis (CF) and pancreatic diseases, is the most recognized cause of fat malabsorption, a variety of additional pathophysiologic mechanisms, such as those seen with critical illness, gastrointestinal surgery, celiac disease, and short bowel syndrome (SBS), may also contribute to fat malabsorption [1,2]. Undernutrition resulting from fat malabsorption in pediatric patients may negatively affect growth and development. The link between undernutrition in young children and child mortality and long-term deficits is well recognized. When a child’s nutritional progress is insufficient, enteral tube feeding may be recommended to provide additional nutrition for growth. The Cystic Fibrosis Foundation (CFF) recommends enteral nutrition (EN) supplementation via a feeding tube to improve age-dependent anthropometrics in patients with poor nutritional status [3]. This approach is utilized for other chronic diseases in order to improve poor linear growth and/or inadequate weight gain. EPI is an underdiagnosed condition in children, in whom enzyme deficiencies may be possible causes of malnutrition and growth failure [4]. Like people with CF (pwCF), pediatric patients with chronic pancreatitis may also experience EPI [5].

RELiZORB (Alcresta Therapeutics, Inc.; Waltham, MA, USA) immobilized lipase cartridge (ILC) is a single-use digestive enzyme cartridge that connects in-line with enteral feeding circuits to hydrolyze triglycerides in enteral formulas (bolus and continuous feeds; [6]). ILC contains small white polymer beads that have lipase attached to their surface. The fat in enteral formula is hydrolyzed as it comes in contact with the lipase in the cartridge. Because triglycerides containing long-chain fatty acids must be hydrolyzed for intestinal absorption, ILC is important for the absorption of these key dietary fatty acids. It was cleared for use in adults in 2015, children 5 years of age and older in 2017, children 2 years of age and older in 2023, and all children, including neonates and infants, in 2025. Recent guidelines for administration of pancreatic enzyme replacement therapy (PERT) with enteral feeding recommend the use of ILC in pwCF and people with fat malabsorption as a result of EPI [7,8]. Guidelines for pediatric patients with chronic pancreatitis recommend pancreatic enzyme replacement per the guidelines in pwCF [5].

Clinical benefits of administering enteral formulas through an ILC have been most widely studied in children and adults with CF. ILC-mediated triglyceride hydrolysis resulted in enhanced absorption of essential fatty acids and was associated with a reduction in gastrointestinal adverse events associated with fat malabsorption and improved growth in children. Use of ILCs increased plasma concentration of omega-3 fatty acids in two short-term studies (≤90 days) in pwCF 5 years of age and older, including adults [9,10]. Also, the severity of most symptoms of malabsorption decreased with 7 [9] or 90 days [10] of ILC use. Two single-center clinical reports also described significant decreases in adverse gastrointestinal events with ILC use in pwCF [11,12].

Two studies evaluated anthropometrics in pwCF using ILCs for 12 months [13,14]. Significant improvements from baseline in estimated mean weight z-scores at 6 and 12 months (0.20 at 6 and 12 months) were reported in patients 2 to 18 years of age (*n* = 93) [13]. The frequency of achieving the 50th percentile for weight and BMI, a nutritional target associated with improved pulmonary outcomes in CF [15], increased steadily from baseline to 12 months: weight percentiles reached the 50th percentile in 18%, 25.5%, and 28.9% of patients at 0, 6, and 12 months, respectively. BMI reached the 50th percentile in 37.1%, 49.1%, and 50.0% of patients at 0, 6, and 12 months, respectively. Additionally, a single-center retrospective case series evaluating ILC use over 12 months showed significant improvements in adjusted mean height z-score (0.25 and 0.27 at 6 and 12 months, respectively) when compared with oral PERT alone in 29 children with CF and EPI (average patient age was 8.4 years, range: 0.5 to 17 years) [14]. Taken together, ILC use was associated with clinical benefit in children with CF. However, limited data have been published regarding the effect of ILC use on growth in infants or children younger than 5 years of age with CF or other causes of EPI or fat malabsorption. The objective of this study was to evaluate real-world growth outcomes of pediatric patients who initiated ILC use between 1 and 4 years of age. Given the rarity of pediatric patients with fat malabsorption requiring EN, analysis of real-world evidence was employed to evaluate potential ILC clinical benefit.

## 2. Materials and Methods

### 2.1. Study Design

This retrospective observational study evaluated real-world data from young children who initiated ILC use when 1 to 4 years of age as part of an enteral feeding program. The data was collected from a third-party reimbursement support program (RELiZORB Support Services, Huntington Beach, CA, USA) database that contains patient medical records collected to support insurance (private and federal and state programs) approval for the use of ILC. The US Food and Drug Administration (FDA) guidance “Use of Real-World Evidence to Support Regulatory Decision-Making for Medical Devices” was followed in the development of this protocol [16].

### 2.2. Study Population

The study population included children who were enrolled in the reimbursement support program, initiated ILC use between October 2019 and October 2023, and were aged 1 year to 4 years 11.99 months. The ILC use start date was defined as the first ILC shipment date if the start date was not reported in available clinic notes. The patient’s healthcare provider determined the indication for ILC use and diagnosis codes, the number of ILCs prescribed per day, the type, daily volume, and method of EN delivery, as well as the cadence of follow-up visits. Patients with a diagnosis code of CF, regardless of additional diagnoses, were grouped as having CF. Given the newly available diagnosis codes in 2023 for SBS, an indication of SBS for ILC use was based on a clinical review of medical history in the clinic notes. Given the nature of the database, availability of anthropometric data was not protocolized but driven by the frequency and timing of patient status requests by insurance providers for reauthorization.

### 2.3. Study Data Collection

Anonymized patient data were collected on study-specific casebooks from electronic files (i.e., PDF files of clinician notes used to support reimbursement efforts) that are stored in the reimbursement database. Data was collected from October 2019 through October 2024 for patients who initiated ILC use between October 2019 and October 2023; up to one year of follow-up growth measurements were included for each patient. Because of a one-year follow-up period, the database includes data reported when patients were 1 to 5 years of age. Patient compliance with prescribed ILC use and ongoing use at 12 months was estimated by monthly shipment records. Any potential interruptions in ILC use were not taken into account. If ILC use was discontinued, the end date was based on the final ILC shipment date. The reason for discontinuation was determined by reviewing medical records and case manager notes. Data was independently reviewed and underwent 100% quality control checks to ensure accurate transcription from clinic notes. Duration of EN use prior to ILC was estimated by gastrostomy-tube placement date and the first ILC shipment date.

### 2.4. Study Endpoints

The primary efficacy endpoint was the change from baseline in body weight z-score at 12 months based on the Centers for Disease Control and Prevention (CDC) growth charts. Secondary efficacy endpoints included change in BMI (z-scores and percentiles) at 12 months and change in weight z-scores at 3, 6, 9, and 12 months. Exploratory efficacy endpoints included change in CDC height z-score after 3, 6, 9, and 12 months of ILC use. Change in weight-for-length in children less than 2 years of age was a post hoc analysis. The CDC and the American Academy of Pediatrics recommend the use of World Health Organization (WHO) growth curves for children less than 24 months of age and CDC growth curves for children aged 2 to 19 years. CDC growth curves were used for standardization of all weight and length/height measures. WHO growth curves were used to determine weight-for-length z-scores and percentiles for patients initiating ILC use when 1 year of age, and CDC growth curves were used to determine BMI z-scores and percentiles for patients initiating ILC use when 2 to 4 years of age.

Baseline growth measurements were taken between 3 months before and up to 7 days after ILC initiation. The 3 month and 7 day window for baseline growth measurements was driven by the timing of the initial request for insurance authorization. Growth measurements at other time points were collected between 1 month prior to and up to 2 months after the end of the 3, 6, 9, or 12-month time period. A missing growth measurement (height, weight, or BMI) was calculated (not imputed) if the other two growth measurements were available. Missing data was not imputed; only data with verifiable source documentation was included in this study in order to meet data suitability criteria required for FDA decision making [16]. Efficacy analyses were conducted on an efficacy analysis population for whom growth measurements were recorded at baseline and at least one follow-up measurement (3, 6, 9, or 12 months). Given that not all patients had follow-up growth measurements at each timepoint, mean baseline values were calculated for each timepoint based on the number of patients contributing data at each timepoint, and the mean was used to determine change from baseline for each timepoint. Previous pilot analyses of similar data, and a published study in 29 pediatric pwCF [14], indicated that a sample size of 50 would be adequate to detect a statistically significant improvement in estimated weight z-score over 12 months of ILC use. Concomitant medication use, any changes to ILC or EN use, and hospitalizations were not transcribed from the clinic notes to the data collection forms, given the incomplete availability of clinic notes to accurately track changes in EN or to evaluate the impact of hospitalizations on growth measures.

Safety was assessed by the incidence of customer complaints, including adverse gastrointestinal events collected as part of the ongoing manufacturer post-market surveillance program. The overall product complaint rate and the rate of gastrointestinal adverse events in children aged 1 to 5 years were compared to the rates in children and adults older than 5 years. The number of devices shipped during the study period was used to calculate the rates of complaints/gastrointestinal adverse events for the two groups.

### 2.5. Statistical Analysis

**Figure 1:**
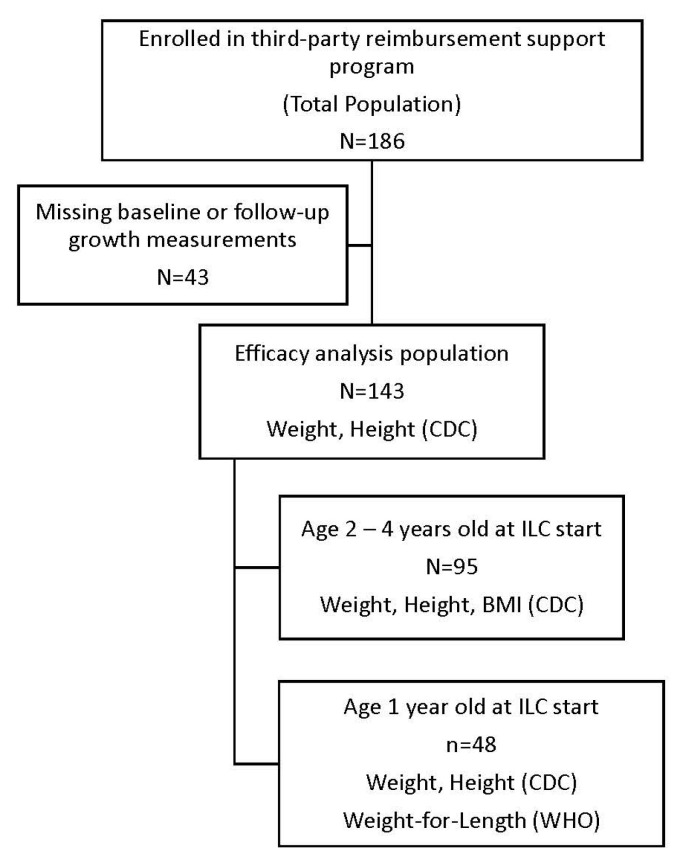
Study populations and data analysis.

All efficacy analyses and descriptive summaries of growth were based on data transcribed from available clinic notes with no imputation for missing data. Weight, height, and BMI were normalized to age- and sex-matched z-scores and percentiles using CDC growth charts [17] or WHO Child Growth Standards [18]. Exploratory post hoc analyses of growth in pwCF and those without CF were performed. To evaluate these results against the CFF goals to achieve BMI/weight-for-length ≥ 50th percentile, weight and BMI percentiles in children 2 years of age and older were calculated for pwCF based on CDC growth curves, and weight and weight-for-length percentiles in children younger than 2 years of age were calculated for pwCF based on WHO growth curves (Figure 1). Efficacy endpoints were analyzed as changes from baseline and tested at 2-sided α = 0.05 using a one-sample *t*-test to take advantage of intra-participant correlation and the resultant reduction in variability. The Wilcoxon signed-rank (WSR) test was prespecified as a supportive robust analysis to be performed irrespective of conformity to normality of the data, which was not assessed formally. No adjustment to the α level was made for multiple tests. The change from baseline in the number of pwCF with weight and BMI/weight-for-length ≥ 50th percentile was tested by the McNemar test. SAS version 9.4 (SAS Institute, Cary, NC, USA) was the main statistical package used for statistical analysis and reporting.

## 3. Results

### 3.1. Patient Population

A total of 186 patients from 90 clinics in the United States (US) were included in this study, with no site contributing more than 4% of the population; 143 patients (efficacy analysis population) had baseline and follow-up growth measurements (Figure 1). Most patients (76% of the efficacy population) were diagnosed with CF (Table 1). Patients in the efficacy population with diagnoses other than CF (*n* = 34) had a wide range of rare diseases, with most patients having SBS (Table 1). This subset of patients had lower mean weight and height z-scores at baseline (−1.8 and −1.9, respectively) than the CF efficacy population (*n* = 109).

Ninety-one percent of the patients had baseline anthropometric measures within 60 days of initiation of ILC use. Baseline anthropometric measure z-scores for total and efficacy populations were all less than the CDC population mean (Table 2). Younger children and those with diagnoses other than CF started with more severe growth deficits. Patients initiating ILC use at 1 year of age had lower mean weight z-scores at baseline than the 2- to 4-year-old population (Table 2). Mean baseline weight and height z-scores for 1-year-olds in the efficacy population based on WHO growth curves (Supplemental Table S1) were also lower than those of the 2- to 4-year-old efficacy population (Table 2). Patient demographics and baseline characteristics of the total population and efficacy population were comparable (Table 1 and Table 2).

### 3.2. Enteral Nutrition and ILC Use

The median gastrostomy-tube placement was 9 months prior to the first shipment of ILC in the 67% of patients with known gastrostomy-tube placement dates. The initial ILC shipment was within 1 month of gastrostomy-tube placement in 29% of the patients. Patients with EPI were also receiving oral PERT and/or feeds mixed with PERT at the time of ILC start. Most patients (82%) received EN as overnight enteral feeds with a median daily volume of 540 mL (range 120 to 1560 mL) using 1 ILC per 500 mL enteral formula (range 1 to 4 ILC/day) at the start of ILC use. A subset of patients (14%) received enteral feeds for 16 to 24 h/day. In the efficacy analysis population, 120 (84%) of 143 patients received a minimum of two-thirds of prescribed ILC shipments, indicating a high rate of compliance with ILC use.

### 3.3. Patient Disposition

Most of the efficacy (80%) and total (69%) populations were using ILCs at the end of the 12-month data collection period. The most common reason for discontinuation of ILC use during the 12-month study period was that the patient no longer received tube feeding (10% and 6% of the total and efficacy populations, respectively). One percent (2 of 186) of patients discontinued ILC use due to documented lack of efficacy.

### 3.4. Efficacy Analysis

**Figure 2:**
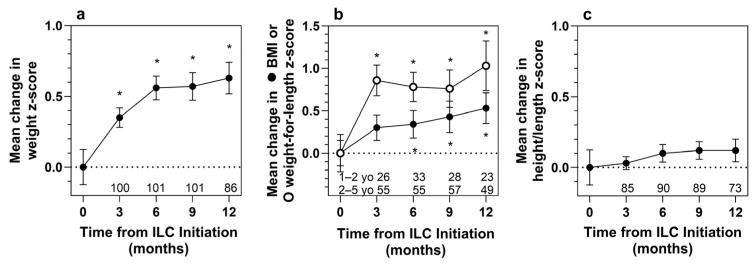
Mean changes in efficacy population z-scores over 12 months of ILC use for (**a**) weight (CDC), (**b**) BMI in patients initiating ILC use when 2 to 4 years old (CDC), and weight-for-length in patients initiating ILC use when 1 year old (WHO), and (**c**) height (CDC). Error bars indicate SEM, and asterisks indicate *p* < 0.05 by *t*-test. Numbers below each datapoint indicate the number of patients with a measurement at that timepoint. BMI, body mass index; ILC, immobilized lipase cartridge; yo, year old.

In the efficacy analysis population, mean weight and BMI z-scores improved from baseline to 12 months after initiation of ILC use (0.63 [*p* < 0.001] and 0.53 [*p* = 0.006], respectively) (Figure 2, Table 3), indicating that weight and BMI increased in the efficacy population at a faster rate than among sex- and age-matched reference standards. Significant improvement in the mean weight z-score was observed after 3 months of ILC use. The increase in mean weight z-score at 12 months was greater in patients who initiated ILC use at 1 year of age than in older patients (1.17 vs. 0.38). The improvement in mean weight z-scores among 1-year-olds at 12 months was smaller when calculated with WHO growth curves (0.72, Supplemental Table S2) than with CDC growth curves (1.17, Table 3). Increases in mean weight z-scores in 1-year-olds at 3, 6, and 9 months were all statistically significant, as were increases in mean weight z-scores in 2- to 4-year-old patients at 3, 6, and 9 months. Significant improvements in the mean change in weight-for-length z-score (WHO) for 1-year-olds and BMI z-score (CDC) in 2- to 4-year-olds were observed at all timepoints. The mean CDC height z-score increased by 0.12 from baseline to month 12 in the efficacy population, but that increase was not statistically significant (*p* = 0.128).

**Figure 3:**
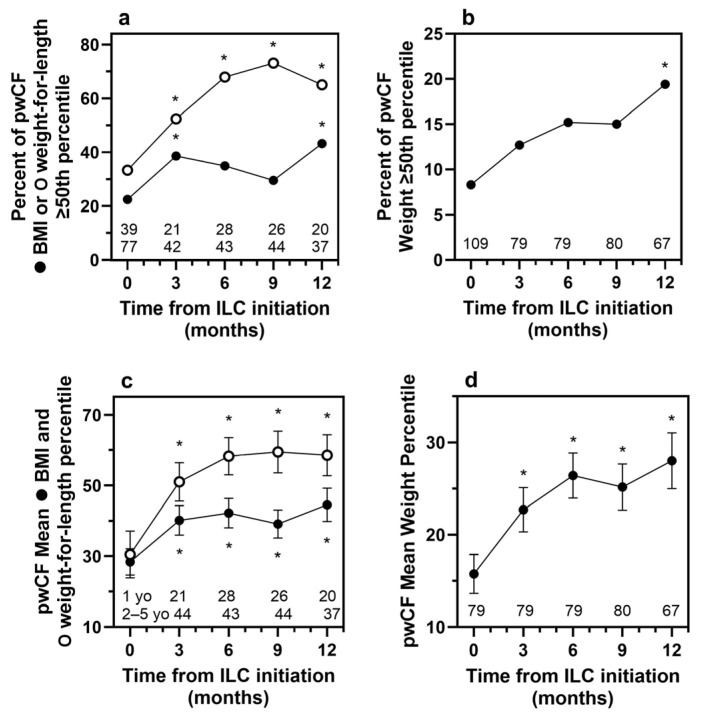
Percent of pwCF at ≥50th percentile for (**a**) BMI in patients initiating ILC when 2 to 4 years of age (● CDC) and weight-for-length in patients initiating ILC when 1 year of age (○ WHO) and (**b**) weight (● CDC). Mean (SEM) percentiles for (**c**) BMI in pwCF initiating ILC when 2 to 4 years of age (● CDC) and weight-for-length in pwCF initiating ILC when 1 year of age (○ WHO), and (**d**) weight (● CDC) in pwCF. Numbers below each datapoint indicate the number of patients with a measurement; the top row of numbers refers to the WHO weight-for-length sample size (1-year-olds). Asterisks indicate statistically significant change from baseline (*p* < 0.05) by McNemar test in panels (**a**,**b**) and by *t*-test in panels (**c**,**d**). BMI, body mass index; CDC, Centers for Disease Control and Prevention; ILC, immobilized lipase cartridge; pwCF, patients with cystic fibrosis; SEM, standard error of the mean; WHO, World Health Organization.

Because the majority of the efficacy population comprised pwCF, the percentage of patients with BMI/weight-for-length or weight, ≥ 50th percentile, as well as changes in weight and BMI in pwCF, were evaluated in a subgroup analysis. Among pwCF, 19% achieved weight ≥ 50th percentile after 12 months of ILC use versus 8% at baseline (*p* = 0.020), and 43% of the patients initiating ILC use when 2 to 4 years of age achieved BMI ≥ 50th percentile versus 22% at baseline (*p* = 0.021, Figure 3a,b). Among the pwCF initiating ILC use when 1 year of age, 65% achieved weight-for-length ≥ 50th percentile after 12 months of ILC use versus 33% at baseline (*p* = 0.020). Improvements in mean weight and BMI/weight-for-length percentiles among pwCF were statistically significant (*p* < 0.05) at all time points (Figure 3c,d). In pwCF, mean weight improved from the 16th percentile at baseline to the 28th at 12 months (*p* < 0.001).

In contrast to the CF population, patients with other conditions had smaller (0.39 and 0.44) improvements in mean weight and BMI z-scores at 12 months, respectively, that were not statistically significant (Table 3). Only three patients diagnosed with SBS had weight data at baseline and 12 months. The improvement in mean height z-score (0.20) in the population without CF was greater than that seen in pwCF (0.11) at 12 months.

### 3.5. Safety Analysis

No patient deaths or any serious injuries associated with ILC use were reported in post-market surveillance at the time of analysis. Post-market surveillance did not identify any unexpected risks or increased risk of adverse events during the study period in the 1- to 5-year-old population compared to older patients. There were 2 and 32 gastrointestinal adverse events reported in the 1 to < 5-year-old and 5-year-old and older populations during the study period, respectively. The incidence of gastrointestinal adverse events was less than 1 per 10,000 devices used in both populations.

## 4. Discussion

The goal of this study was to evaluate real-world data in children who initiated ILC use when 1 to 4 years of age to evaluate the potential impact of ILCs on growth for up to 12 months. Given the relative rarity of pediatric patients with fat malabsorption requiring EN, such that no one center prescribed ILC use to more than 7 children over a 4-year period, these types of multicenter analyses are essential to evaluate potential ILC clinical benefit. Although these results are not based on a well-controlled clinical study, the FDA recognizes that a wealth of real-world data covering medical device experience exists and is routinely collected in the course of treatment and management of patients [16]. This study represents the largest and most geographically diverse cohort to date evaluating real-world ILC usage in patients less than 5 years of age. Previous multicenter and single-center studies included fewer than 30 patients, less than 5 years of age [13,14]. This study had no control group, but the magnitude of the increase in mean weight z-score at 1 year reported in this study (0.63) is larger than that (0.09) in a historical control group of pwCF not using ILCs [13]. This study has demonstrated the largest impact on mean weight and BMI z-scores over a 12-month period observed to date, likely a result of limiting the patient population to younger patients with the greatest growth potential. For most of the study period (prior to August 2023), ILCs were not yet indicated for patients less than 5 years of age. Clinicians may have reserved off-label ILC use for the most underweight patients or those not otherwise responding to PERT. This improvement in mean weight z-scores and percentiles may be due to the increased availability of critical free fatty acids delivered through enteral feeding [9] at a time of particularly important physical development. Additionally, ILC use may be particularly effective in very young children with immature bile acid metabolism, feeding intolerance, or limited active lipase.

Although most of the patients in this study were diagnosed with CF (76% of the efficacy population), the pwCF in this report were younger and more underweight (mean weight z-score at baseline −1.5, 16th percentile) than pwCF studied in previous reports of ILC use [11,12,13,14]. In a previous study [13], 18 patients, 2 to 5 years of age, had a mean BMI at the 41st percentile compared to the 32nd percentile in 2- to 4-year-olds in this report.

In contrast to previous studies in pwCF from Sathe [13] and Shrivastava [14], statistically significant improvements in length or height z-scores were not observed in this study. Although this real-world evidence study included less length and height data compared to the amount of weight measurements, additional real-world evidence would likely not affect the magnitude of the change in mean height z-scores at 6 months (0.07), which was lower than estimated means observed by Sathe (0.17) [13] and Shrivistava (0.25) [14] over the same time period. As noted previously by Shrivistava [14], weight tends to increase prior to height in underweight pwCF who begin to receive adequate nutrition [19].

Patients with indications other than CF, of which SBS was the most common, represented 24% of the efficacy population (*n* = 34 patients). This small subgroup size limited the ability to draw conclusions regarding the greater improvement in mean height z-score (0.20) compared to that of pwCF (0.11) and the modest improvement in mean weight z-scores (0.39) compared to pwCF (0.69) over 12 months. The minimal effect of ILC use on height in children with CF, compared to patients without CF, may be due to intrinsic differences in pwCF [19]; other studies of therapeutic and nutritional interventions reported a greater impact on weight than height in pwCF [20]. Information regarding concomitant parenteral nutrition supplementation was not collected in this study, so we were unable to determine if there were any changes in parenteral supplementation as a result of ILC use. Transition from PN to EN in patients with SBS may have been a priority in some patients, and improvements in weight z-score would have been a secondary objective.

The CFF recommends that children with CF achieve and maintain a BMI ≥ 50th percentile [15,21]. Clinically meaningful and statistically significant improvements in the number of pwCF reaching this important milestone by 12 months, as well as weight ≥ 50th percentile by 3 months, were observed after initiating ILC use in our patient sample. The percentage of 1-year-old pwCF with weight-for-length ≥ 50th percentile also increased over the course of 12 months, with statistically significant improvement as early as 3 months. Collaboration among a multidisciplinary team, including registered dieticians, to evaluate and aggressively manage pediatric patients with EPI may have contributed to the successful outcomes observed in this real-world study [22].

Baseline characteristics of the total and efficacy populations were generally comparable, suggesting that growth outcomes observed in the efficacy population can be generalized to the broader population of pwCF and other patients with fat malabsorption as a result of EPI (e.g., pancreatitis) or SBS. The total population in this report includes the vast majority of children aged 1 to 4 years old who have been prescribed ILCs since 2019. There is limited data availability prior to 2019 as a result of incomplete data from earlier reimbursement support program databases. It is estimated that fewer than 10% of patients received ILCs through hospital-owned specialty pharmacies and are not represented in the database.

Post-market surveillance did not identify any unexpected risks in the 1- to 5-year-old age group. No new risks associated with ILC use were identified in this patient population. The almost daily and continued ILC use, as evidenced by consistent monthly shipments of ILC as well as ongoing use in the total and efficacy populations, is indirect evidence of the safety and tolerability of this device. Because product complaints are reported voluntarily, it is not always possible to reliably estimate the frequency of adverse events or establish a causal relationship with ILC exposure. It should be noted that industry standard dictates that all contractors, including the third party managing the reimbursement support program, are required to report to the manufacturer if they become aware of any serious injuries associated with the device when reviewing clinic notes during the insurance authorization process. Therefore, any serious injuries associated with ILC use documented in clinic notes would have been reported to the manufacturer and included in the product complaint database.

There are several limitations to this study. The nature of the data source resulted in missing information that would have been helpful in interpreting these findings. Information regarding growth measures in the year prior to ILC use, hospitalizations, potential changes in gastrointestinal symptoms, vitamin or fatty acid deficiency, EN, PERT, and other concomitant medication use was only intermittently available in the reimbursement support program clinic notes.

This was a single-cohort study of real-world data without a control group, which limits the ability to interpret the efficacy results. Some patients in this study initiated ILC use within a month of initiating enteral feeding, which is not unexpected for patients with low weight z-scores and EPI symptoms, but it does limit the conclusions regarding ILC use. Data collected during clinical care or in the home setting may not have the same quality controls as data collected within a clinical trial setting. Additionally, as the study period included the 2020 COVID-19 pandemic, many patients were seen in virtual clinic visits that precluded the routine collection of weight and height measurements.

There may have been selection bias introduced as the patient population was limited to those seeking insurance coverage for ILC. A few states in the US (e.g., South Carolina and Alabama) did not cover ILC in their Medicaid program at the time of this study, so only patients with private insurance from those states were included. Insurance approval was granted for the vast majority of patients. The likelihood of this limitation affecting the generalizability of these results is small, given that the data were collected from 90 sites across the US.

Conclusions regarding ILC use in children less than 1 year of age and those without CF require further investigation. In the subpopulation without CF, there were modest improvements in weight and height z-score that did not reach statistical significance, likely due to the smaller sample size (*n* = 34). Ongoing investigations include a manufacturer-sponsored registry of ILC use in pediatric patients with intestinal failure [23] and a 90-day trial evaluating ILC efficacy in parenteral nutrition-dependent children with SBS [24].

It is difficult to determine the underlying factors leading to the plateau in weight and BMI z-scores, as changes to patient nutrition and hospitalizations during the study period were not collected. However, a plateau after a significant increase in weight following initiation of nutritional support is common and believed to be a result of metabolic adaptation.

As noted in the study limitations, the use of CF transmembrane conductance regulator (*CFTR*) modulator drugs during the observation period was not collected in this study. *CFTR* modulators were approved by the FDA in February 2017 (ivacaftor), August 2018 (lumacaftor and ivacaftor), and April 2023 (elexacaftor, tezacaftor, and ivacaftor [ETI]) for use in a subset of pwCF 2 years of age and older. Ivacaftor received subsequent FDA approval for use in a subset of pwCF > 6 months of age in January 2020, and lumacaftor and ivacaftor gained FDA approval for use in a subset of pwCF 1 year of age in September 2022. Therefore, concomitant ETI use may have taken place in the last 6 months of the 48-month eligibility window for this study (last patient-initiated ILC use in October 2023). Improvements in weight and BMI have been reported in children taking *CFTR* modulators, with improved fat absorption part of the underlying mechanism [25,26]. More robust improvements in BMI have been reported with highly effective *CFTR* modulators (ivacaftor in patients with gating mutations and ETI) compared to dual combination therapies (lumacaftor and ivacaftor, and tezacaftor and ivacaftor) [26]. However, pancreatic insufficiency has not been demonstrated to resolve in most children, suggesting a continued need for exogenous lipase [27,28]. Taken together, while concomitant use of *CFTR* modulators may have enhanced the impact of ILC in a small portion of our population (estimated that 20% of our population receives *CFTR* modulators), it is unlikely to have had a significant influence on our results.

In a 24-week study of ETI use in 75 pediatric (2 to 5 years of age) pwCF with at least one *CFTR* gene F508del allele, the mean baseline BMI z-score was normal (0.09), and the change in BMI z-score following 24 weeks of ETI treatment was 0.1 [29]. By contrast, the magnitude of BMI z-score improvement observed in this study’s 2- to 4-year-old population following 6 months of enteral formula administration through ILC was 0.34. It is unlikely that these newer therapies are solely responsible for the magnitude of growth improvements reported here. Additionally, the greatest improvement in mean weight z-score in this study was observed in patients who initiated ILC use at 1 year of age, an age not yet approved for ETI use during the study period.

It is important for the treating clinician to be aware that EPI and fat malabsorption extend beyond pancreatic dysfunction or surgery [1,2,3,4,30]. Early diagnosis of fat malabsorption and early nutritional intervention in children, including ILC use with EN, may improve growth outcomes.

## 5. Conclusions

Nutritional care and support are an integral part of management for pwCF and other malabsorption-related conditions. Based on this real-world evidence, EN administered through an ILC in young children 1 to 5 years old and predominantly with CF was associated with significant improvements in weight and BMI z-scores. No new risks or an increased risk of adverse events were identified in this patient population. Taken together, the evidence supports the safety and tolerability of ILC use in this population and suggests that ILC may be a beneficial component of a successful EN regimen for the 1- to 5-year-old population with EPI. Conclusions regarding ILC use in children less than 1 year of age and those without CF require further investigation. Future controlled studies evaluating clinical outcomes associated with ILC use in children less than 1 year of age, pwCF, and with other malabsorptive conditions such as SBS are warranted.

## Figures and Tables

**Table 1:** Demographics.

Characteristic	All Patients (*n* = 186)	Efficacy Population (*n* = 143)
Sex, *n* (%)		
Male	100 (54%)	76 (53%)
Female	86 (46%)	67 (47%)
Age, years, mean (SD)	2.9 (1.2)	2.9 (1.2)
Min, Max	1.0, 4.99	1.0, 4.99
Age, years, *n* (%)		
1	63 (34%)	48 (34%)
2	36 (19%)	28 (20%)
3	42 (23%)	28 (20%)
4	45 (24%)	39 (27%)
Referral diagnosis, *n* (%)		
Cystic fibrosis (CF)	128 (69%)	109 (76%)
Other diagnosis *	58 (31%)	34 (24%)
Short bowel syndrome	24 (13%)	14 (10%)
Exocrine pancreatic insufficiency in patients without a diagnosis of CF	8 (4%)	5 (4%)
Pancreatitis	2 (1%)	2 (1%)
Failure to thrive	2 (1%)	1 (1%)

* Indications for use reported in >1 patient. Additional indications for ILC use in 1 patient include Alagille syndrome, CHARGE syndrome, complications of intestinal transplant, congenital diffuse hyperinsulinemia, end-stage renal disease, eosinophilic esophagitis, feeding difficulties, gastrocutaneous fistula due to gastrostomy-tube, generalized intestinal dysmotility, hereditary sideroblastic anemia, intestinal failure, intestinal transplant, malnutrition, mitochondrial metabolism disorder, mosaic trisomy 9, neuroblastoma, pancreatectomy, pancreatic disease, persistent vomiting, porto-systemic shunt, Shwachman–Diamond syndrome, and Whipple procedure. CF, cystic fibrosis; SD, standard deviation.

**Table 2:** Baseline anthropometric measures.

Measure		All Patients (*n* = 186)	Efficacy Population		
			All 1- to 4-Year-Olds (*n* = 143)	1-Year-Olds (*n* = 48)	2- to 4-Year-Olds (*n* = 95)
Weight	*n*	172	143	48	95
CDC z-score	Mean (SD)	−1.6 (1.4)	−1.6 (1.3)	−2.3 (1.3)	−1.2 (1.2)
CDC percentile	Mean (SD)	17.3 (22.3)	16.7 (21.5)	7.0 (12.8)	21.6 (23.4)
Height	*n*	166	139	47	92
CDC z-score	Mean (SD)	−1.3 (1.5)	−1.2 (1.3)	−1.6 (1.4)	−1.1 (1.2)
CDC percentile	Mean (SD)	23.1 (24.8)	22.9 (24.4)	17.4 (22.9)	25.7 (24.9)
BMI	*n*	105	90	47	90
CDC z-score	Mean (SD)	−0.7 (1.2)	−0.7 (1.1)	NA	−0.7 (1.1)
CDC percentile	Mean (SD)	31.7 (28.3)	32.9 (27.6)	NA	32.9 (27.6)
Weight-for-length	*n*			47	
WHO z-score	Mean (SD)	NA	NA	−0.7 (1.0)	NA
WHO percentile	Mean (SD)	NA	NA	30.8 (26.9)	NA

NA, not applicable.

**Table 3:** Growth measurement summary and change over time at months 3, 6, 9, and 12 in patients initiating ILC use for the efficacy population and subpopulations.

Measure	Statistic	Months Following ILC Initiation			
		3 Months	6 Months	9 Months	12 Months
Efficacy Population (*n* = 143)					
Weight	*n*	100	101	101	86
Baseline z-score	Mean (SD)	−1.58 (1.24)	−1.57 (1.34)	−1.57 (1.30)	−1.63 (1.42)
z-score at month	Mean (SD)	−1.24 (1.19)	−1.01 (1.17)	−1.00 (1.17)	−1.01 (1.37)
z-score change	Mean (SD)	0.35 (0.69)	0.56 (0.85)	0.57 (0.98)	0.63 (1.03)
	*t*-test *p*-value	<0.001	<0.001	<0.001	<0.001
	WSR *p*-value	<0.001	<0.001	<0.001	<0.001
Height	*n*	85	90	89	73
Baseline z-score	Mean (SD)	−1.13 (1.15)	−1.12 (1.22)	−1.07 (1.16)	−1.19 (1.33)
z-score at month	Mean (SD)	−1.09 (1.11)	−1.02 (1.22)	−0.95 (1.15)	−1.07 (1.33)
z-score change	Mean (SD)	0.03 (0.43)	0.10 (0.59)	0.12 (0.59)	0.12 (0.69)
	*t*-test *p*-value	0.501	0.104	0.063	0.128
	WSR *p*-value	0.793	0.146	0.082	0.093
BMI (2 to 4 years old)	*n*	55	55	57	49
Baseline z-score	Mean (SD)	−0.70 (1.20)	−0.74 (1.07)	−0.82 (1.06)	−0.70 (1.12)
z-score at month	Mean (SD)	−0.40 (1.18)	−0.40 (1.13)	−0.39 (1.11)	−0.18 (1.16)
z-score change	Mean (SD)	0.30 (1.12)	0.34 (1.20)	0.43 (1.40)	0.53 (1.28)
	*t*-test *p*-value	0.053	0.038	0.024	0.006
	WSR *p*-value	0.040	0.016	0.047	0.007
1 year old (*n* = 48)					
Weight	*n*	33	38	32	27
Baseline z-score	Mean (SD)	−2.36 (1.19)	−2.31 (1.34)	−2.22 (1.23)	−2.55 (1.41)
z-score at month	Mean (SD)	−1.73 (1.23)	−1.32 (1.20)	−1.11 (1.12)	−1.38 (1.55)
z-score change	Mean (SD)	0.63 (0.63)	0.99 (0.84)	1.11 (0.88)	1.17 (1.07)
	*t*-test *p*-value	<0.001	<0.001	<0.001	<0.001
	WSR *p*-value	<0.001	<0.001	<0.001	<0.001
Height	*n*	26	33	28	24
Baseline z-score	Mean (SD)	−1.53 (1.23)	−1.53 (1.37)	−1.36 (1.16)	−1.58 (1.57)
z-score at month	Mean (SD)	−1.51 (1.21)	−1.44 (1.30)	−1.01 (1.08)	−1.40 (1.79)
z-score change	Mean (SD)	0.02 (0.53)	0.09 (0.64)	0.34 (0.70)	0.18 (1.53)
	*t*-test *p*-value	0.858	0.418	0.015	0.397
	WSR *p*-value	0.853	0.511	0.015	0.419
Weight-for-length	*n*	26	33	28	23
Baseline z-score	Mean (SD)	−0.79 (1.13)	−0.59 (1.03)	−0.56 (1.09)	−0.90 (1.21)
z-score at month	Mean (SD)	0.07 (0.78)	0.18 (1.02)	0.20 (1.04)	0.13 (0.87)
z-score change	Mean (SD)	0.86 (0.92)	0.78 (0.99)	0.76 (1.17)	1.03 (1.41)
	*t*-test *p*-value	<0.001	<0.001	0.002	0.002
	WSR *p*-value	<0.001	<0.001	0.002	0.001
2 to 4 years old (*n* = 95)					
Weight	*n*	67	63	69	59
Baseline z-score	Mean (SD)	−1.20 (1.08)	−1.13 (1.13)	−1.27 (1.23)	−1.22 (1.22)
z-score at month	Mean (SD)	−0.99 (1.10)	−0.83 (1.11)	−0.95 (1.19)	−0.84 (1.26)
z-score change	Mean (SD)	0.21 (0.67)	0.31 (0.76)	0.32 (0.93)	0.38 (0.92)
	*t*-test *p*-value	0.014	0.002	0.006	0.003
	WSR *p*-value	0.011	0.002	0.015	0.006
Height	*n*	59	57	61	49
Baseline z-score	Mean (SD)	−0.95 (1.08)	−0.88 (1.07)	−0.93 (1.14)	−1.01 (1.17)
z-score at month	Mean (SD)	−0.91 (1.03)	−0.78 (1.10)	−0.92 (1.19)	−0.91 (1.01)
z-score change	Mean (SD)	0.04 (0.39)	0.11 (0.57)	0.01 (0.50)	0.10 (0.47)
	*t*-test *p*-value	0.465	0.153	0.840	0.153
	WSR *p*-value	0.841	0.211	0.818	0.285
Patients with Cystic Fibrosis (*n* = 109)					
Weight	*n*	79	79	80	67
Baseline z-score	Mean (SD)	−1.54 (1.19)	−1.46 (1.18)	−1.55 (1.19)	−1.55 (1.27)
z-score at month	Mean (SD)	−1.11 (1.11)	−0.90 (1.05)	−0.95 (1.03)	−0.86 (1.07)
z-score change	Mean (SD)	0.42 (0.58)	0.56 (0.71)	0.60 (0.98)	0.69 (1.00)
	*t*-test *p*-value	<0.001	<0.001	<0.001	<0.001
	WSR *p*-value	<0.001	<0.001	<0.001	<0.001
Height	*n*	67	72	73	58
Baseline z-score	Mean (SD)	−0.94 (1.04)	−0.91 (1.07)	−0.97 (1.05)	−0.96 (1.08)
z-score at month	Mean (SD)	−0.95 (1.00)	−0.84 (1.03)	−0.85 (1.06)	−0.85 (1.03)
z-score change	Mean (SD)	0 (0.37)	0.07 (0.55)	0.12 (0.59)	0.11 (0.69)
	*t*-test *p*-value	0.965	0.289	0.095	0.252
	WSR *p*-value	0.725	0.358	0.095	0.260
BMI (2 to 4 years old)	*n*	44	43	44	37
Baseline z-score	Mean (SD)	−0.85 (1.10)	−0.80 (1.02)	−0.89 (1.07)	−0.75 (1.03)
z-score at month	Mean (SD)	−0.36 (1.00)	−0.29 (0.97)	−0.39 (1.02)	−0.20 (0.96)
z-score change	Mean (SD)	0.49 (0.91)	0.51 (1.00)	0.49 (1.35)	0.55 (1.23)
	*t*-test *p*-value	0.001	0.002	0.019	0.009
	WSR *p*-value	<0.001	0.001	0.013	0.005
Patients with an indication other than CF (*n* = 34)					
Weight	*n*	21	22	21	19
Baseline z-score	Mean (SD)	−1.75 (1.40)	−1.99 (1.76)	−1.62 (1.69)	−1.93 (1.85)
z-score at month	Mean (SD)	−1.69 (1.40)	−1.41 (1.48)	−1.19 (1.60)	−1.53 (2.07)
z-score change	Mean (SD)	0.06 (0.96)	0.58 (1.26)	0.43 (1.01)	0.39 (1.14)
	*t*-test *p*-value	0.779	0.042	0.063	0.150
	WSR *p*-value	0.854	0.047	0.200	0.294
Height	*n*	18	18	16	15
Baseline z-score	Mean (SD)	−1.81 (1.34)	−1.98 (1.45)	−1.51 (1.51)	−2.12 (1.80)
z-score at month	Mean (SD)	−1.65 (1.35)	−1.74 (1.63)	−1.39 (1.46)	−1.92 (1.94)
z-score change	Mean (SD)	0.16 (0.63)	0.23 (0.73)	0.12 (0.60)	0.20 (0.72)
	*t*-test *p*-value	0.299	0.194	0.435	0.296
	WSR *p*-value	0.304	0.181	0.562	0.188
BMI (2 to 4 years old)	*n*	11	12	13	12
Baseline z-score	Mean (SD)	−0.08 (1.42)	−0.54 (1.25)	−0.58 (1.03)	−0.55 (1.39)
z-score at month	Mean (SD)	−0.56 (1.78)	−0.81 (1.58)	−0.36 (1.41)	−0.11 (1.67)
z-score change	Mean (SD)	−0.48 (1.56)	−0.27 (1.66)	0.22 (1.60)	0.44 (1.49)
	*t*-test *p*-value	0.333	0.584	0.635	0.329
	WSR *p*-value	0.083	0.470	0.787	0.424

Patients with growth measures at both baseline and the visit analyzed. All z-scores are based on CDC growth charts except for weight-for-length in the 1-year-old population, which is based on WHO growth charts. BMI, body mass index; CDC, Centers for Disease Control and Prevention; SD, standard deviation; WHO, World Health Organization; WSR, Wilcoxon signed-rank.

## Data Availability

The dataset presented in this article is not available due to privacy restrictions.

## Supplementary Materials

The following supporting information can be downloaded at https://www.mdpi.com/article/10.3390/nu18020287/s1, Table S1: Baseline anthropometric measures (WHO) in patients in the efficacy population initiating ILC use when 1 year of age; Table S2: Growth measurement summary and change over time at months 3, 6, 9, and 12 in patients in the efficacy population initiating ILC use when 1 year of age (±Diagnosis of CF).

## Abbreviations

The following abbreviations are used in this manuscript:

BMI	Body mass index
CDC	US Centers for Disease Control and Prevention
CF	Cystic fibrosis
CFF	Cystic Fibrosis Foundation
CFTR	CF transmembrane conductance regulator
EN	Enteral nutrition
EPI	Exocrine pancreatic insufficiency
ETI	Elexacaftor, tezacaftor, and ivacaftor
FDA	US Food and Drug Administration
ILC	Immobilized lipase cartridge
NA	Not applicable
PERT	Pancreatic enzyme replacement therapy
pwCF	People with cystic fibrosis
SBS	Short bowel syndrome
SD	Standard deviation
SEM	Standard error of the mean
US	United States of America
WHO	World Health Organization
WSR	Wilcoxon signed-rank
yo	Year old
