# Supplementary material for: Real-World Evidence of Growth Improvement in Children 1 to 5 Years of Age Receiving Enteral Formula Administered Through an Immobilized Lipase Cartridge
Source: Nutrients. 2026 Jan 16;18(2):287. doi: 10.3390/nu18020287 (PMC12844636; doi:10.3390/nu18020287)
Supplement: Supplementary file 1 [file nutrients-18-00287-s001.zip › nutrients-4078447-supplementary.pdf]

Supplemental Table S1. Baseline anthropometric measures (WHO) in patients in the efficacy population initiating ILC use when 1 year of age. Eighty-three percent (40/48) of the patients were diagnosed with cystic fibrosis.

**Measure**			**1-Year Olds (N=48)**
Weight		N	48
	WHO z-score	Mean (SD)	-1.5 (1.2)
	WHO percentile	Mean (SD)	15.9 (19.4)
Height		N	47
	WHO z-score	Mean (SD)	-1.9 (1.6)
	WHO percentile	Mean (SD)	14.5 (22.6)
BMI		N	47
	WHO z-score	Mean (SD)	-0.4 (1.1)
	WHO percentile	Mean (SD)	38.9 (29.2)

BMI, Body mass index; SD, standard deviation; WHO, World Health Organization.

Table S2. Growth measurement summary and change over time at months 3, 6, 9, and 12 in in patients in the efficacy population initiating ILC use when 1 year of age (± Diagnosis of CF). All z-scores are based on WHO growth charts.

Measure	Statistic	Months following ILC initiation			
		3 months	6 months	9 months	12 months
**1-Year Olds**					
Weight	N	33	38	32	27
Baseline z-score	Mean (SD)	-1.55 (1.08)	-1.51 (1.22)	-1.43 (1.10)	-1.73 (1.28)
z-score at Month	Mean (SD)	-1.06 (1.10)	-0.81 (1.08)	-0.70 (0.97)	-1.00 (1.28)
z-score change	Mean (SD)	0.49 (0.58)	0.71 (0.74)	0.73 (0.80)	0.72 (0.92)
	t-test P-value	<0.001	<0.001	<0.001	<0.001
	WSR P-value	<0.001	<0.001	<0.001	<0.001
Length	N	26	33	28	24
Baseline z-score	Mean (SD)	-1.87 (1.40)	-1.87 (1.57)	-1.68 (1.29)	-1.91 (1.80)
z-score at Month	Mean (SD)	-1.88 (1.36)	-1.76 (1.34)	-1.34 (1.17)	-1.65 (1.68)
z-score change	Mean (SD)	-0.01 (0.60)	0.11 (0.73)	0.34 (0.77)	0.26 (1.04)
	t-test P-value	0.925	0.388	0.029	0.235
	WSR P-value	0.912	0.425	0.040	0.171
Weight-for-length	N	26	33	28	23
Baseline z-score	Mean (SD)	-0.79 (1.13)	-0.59 (1.03)	-0.56 (1.09)	-0.90 (1.21)
z-score at Month	Mean (SD)	0.07 (0.78)	0.18 (1.02)	0.20 (1.04)	0.13 (0.87)
z-score change	Mean (SD)	0.86 (0.92)	0.78 (0.99)	0.76 (1.17)	1.03 (1.41)
	t-test P-value	<0.001	<0.001	0.002	0.002
	WSR P-value	<0.001	<0.001	0.002	0.001
BMI	N	26	33	28	23
Baseline z-score	Mean (SD)	-0.47 (1.18)	-0.28 (1.11)	-0.30 (1.14)	-0.62 (1.28)
z-score at Month	Mean (SD)	0.41 (0.82)	0.47 (1.10)	0.40 (1.07)	0.39 (0.90)
z-score change	Mean (SD)	0.89 (1.01)	0.76 (1.14)	0.70 (1.23)	1.00 (1.52)
	t-test P-value	<0.001	<0.001	0.005	0.004
	WSR P-value	<0.001	<0.001	0.006	0.003
**1-Year Olds with Cystic Fibrosis**					
Weight	N	27	31	39	23
Baseline z-score	Mean (SD)	-1.48 (1.08)	-1.27 (1.07)	-1.35 (1.06)	-1.56 (1.16)
z-score at Month	Mean (SD)	-0.96 (1.11)	-0.67 (1.05)	-0.62 (0.97)	-0.76 (1.05)
z-score change	Mean (SD)	0.53 (0.46)	0.60 (0.61)	0.74 (0.78)	0.80 (0.90)
	t-test P-value	<0.001	<0.001	<0.001	<0.001
	WSR P-value	<0.001	<0.001	<0.001	<0.001
Weight-for-length	N	21	28	26	20
Baseline z-score	Mean (SD)	-0.70 (1.13)	-0.54 (1.07)	-0.60 (1.11)	-0.89 (1.21)
z-score at Month	Mean (SD)	0.00 (0.82)	0.22 (0.95)	0.24 (1.04)	0.27 (0.81)
z-score change	Mean (SD)	0.70 (0.78)	0.76 (1.03)	0.84 (1.11)	1.16 (1.28)
	t-test P-value	<0.001	<0.001	<0.001	<0.001
	WSR P-value	<0.001	<0.001	<0.001	<0.001

BMI, Body mass index; CDC, Centers for Disease Control and Prevention; CF, cystic fibrosis; SD, standard deviation; WHO, World Health Organization; WSR, Wilcoxon signed-rank.
